# Wound healing in weaning, adult, and old rats with provoked incisional hernias. A comparative study

**DOI:** 10.1016/j.clinsp.2022.100106

**Published:** 2022-09-19

**Authors:** Raphael Nogueira do Amaral, Ana Cristina Aoun Tannuri, Junia Marielle Teixeira Rodrigues Neri, Hugo de Souza Reis, Josiane Oliveira Gonçalves, Suellen Serafini, Uenis Tannuri

**Affiliations:** a Medical Researcher, Pediatric Surgery Division, Pediatric Liver Transplantation Unit and Laboratory of Research in Pediatric Surgery (LIM 30), Faculdade de Medicina da Universidade de São Paulo, São Paulo, SP, Brazil; b Professor, Pediatric Surgery Division, Pediatric Liver Transplantation Unit and Laboratory of Research in Pediatric Surgery (LIM 30), Faculdade de Medicina da Universidade de São Paulo, São Paulo, SP, Brazil; c Pediatric Surgery Division, Pediatric Liver Transplantation Unit and Laboratory of Research in Pediatric Surgery (LIM 30), Faculdade de Medicina da Universidade de São Paulo, São Paulo, SP, Brazil; d Biologist, Pediatric Surgery Division, Pediatric Liver Transplantation Unit and Laboratory of Research in Pediatric Surgery (LIM 30), Faculdade de Medicina da Universidade de São Paulo, São Paulo, SP, Brazil; e Head Professor, Pediatric Surgery Division, Pediatric Liver Transplantation Unit and Laboratory of Research in Pediatric Surgery (LIM 30), Faculdade de Medicina da Universidade de São Paulo, São Paulo, SP, Brazil

## Abstract

**Background:**

Incisional hernias are more frequent in adults than in children. It is hypothesized that a more efficient healing process in pediatric patients could explain this difference in incidence. Certain elements of healing such as neovascularization, degree of inflammation, percentage of mature and immature collagen, the proliferation of fibroblasts, and expression of certain genes could explain why healing in children is more efficient when compared to the adult and elderly populations.

**Materials and methods:**

Seventy-one rats of 3 different age groups (weaning, adult, and old) underwent surgery with 3 different incisions (vertical, oblique, and horizontal). During the procedure, the skin and abdominal wall of the animal were sectioned and only the skin was sutured to mimic incisional hernia in the animals. Four weeks after surgery, the rats were euthanized, their skin was removed, and the extent of scar tissue formed in the muscle opening was measured. In addition, samples of the scar tissue were collected for histological, immunohistochemical, and molecular analyzes. Nine rats served as controls.

**Results:**

Shorter-length hernias were formed in weaning rats when compared to old ones when the surgical incision was horizontal (*p* = 0.03). There was a greater proliferation of fibroblasts in rats in the younger age groups, regardless of the type of incision. The Lox gene was more expressed in weaning rats with vertical and oblique incisions.

**Conclusions:**

These differences could explain the better healing and lower incidence of hernias in the pediatric population, although this aspect requires further studies.

## Highlights

• Lower incidence of incisional hernias in pediatric patients.
• Greater proliferation of fibroblasts in younger rats.
• Lox gene is more expressed in weaning rats.

Incisional hernia is a condition that affects patients who undergo surgical procedures, especially in the abdominal region, and subsequently presents an opening in the muscle layer allowing the passage of organs and other internal tissues, without exceeding the skin limit.^1^ There are several risk factors for this type of hernia, such as inadequate suture, infection in the site of incision, vertical rather than horizontal incisions, smoking, age, malnutrition, and obesity.^2^, ^3^, ^4^ All these factors are associated with inefficient healing, a key determining factor for incisional hernia.

These hernias usually affect adults but are not so common in the pediatric population. A study conducted at a Japanese hospital with 2049 patients aged less than 15 years who underwent surgery reported an overall rate of 0.68% of incisional hernia, an incidence rate significantly different from that of adults, estimated at 5% to 50%.^5^ Despite the knowledge about incidences in each age group, few articles address the outcome of incisional hernias in children. Studies comparing the formation mechanism of this type of hernia between different age groups are also very limited. It is hypothesized that wound healing is the key element behind these different incidences of incisional hernia between adults and children, which is the focus of the present study.

Collagen has a major role in the wound healing process, being the main element of the new cellular matrix and responsible for the ability of the new tissue to resist tension.^6^^,^^7^ Other elements of healing such as fibroblast proliferation, neovascularization, degree of inflammation, and expression of certain genes are also shown to be crucial for proper healing.^7^ In addition, some important genes participate in wound healing. The Lox (lysyl oxidase) gene stands out in this process, being responsible for stabilizing collagen.^8^^,^^9^

The histological and genetic mechanisms behind the lower incidence of incisional hernia in the pediatric vs. adult population are still unknown. The objective of the present study was to investigate and analyze these crucial elements in the scar tissue of rats of 3 different age groups using histological, immunohistochemical, and molecular analyzes. This study also sought to understand how incisions in different orientations can interfere with the formation of incisional hernias in rats.

## Materials and methods

The animals were cared for following the criteria set forth in the “Guide for Care and Use of Laboratory Animals” from the National Academy of Sciences. The study protocol was reviewed and approved by the Animal Ethics Committee of the present study's institution.

Seventy-one rats of 3 different ages (weaning: from 23 to 26 days; adults: from 41 to 47 days; old rats: 6 months) were subjected to abdominal incisions in 3 different orientations and nine additional rats (with the same age of experimental animals) that did not undergo any procedure were used as controls. The control animals had the same age as the experimental animals. Table 1 shows the distribution of the groups. Before the procedure, the animals were anesthetized with 30 mg/kg of ketamine hydrochloride (Ketalar®) and 10 mg/kg of dexmedetomidine (Precedex®). For animals in the vertical and oblique incision groups, the length of the skin and abdominal wall muscle incision corresponded to 25% of the size of the animal (disregarding the tail). For animals in the horizontal incision group, the incision length corresponded to 20% of the size of the animal.

**Table 1:** Group distribution according to age, type of incision and number of animals.

Groups	Age	Incision orientation	n
Weaning Vertical (WV)	23 ‒ 26 days	Vertical	8
Adult Vertical (AV)	41 ‒ 47 days	Vertical	8
Old Vertical (OV)	6 months	Vertical	8
Weaning Oblique (WO)	23 ‒ 26 days	Oblique	8
Adult Oblique (AO)	41 ‒ 47 days	Oblique	7
Old Oblique (OO)	6 months	Oblique	8
Weaning Horizontal (WH)	23 ‒ 26 days	Horizontal	8
Adult Horizontal (AH)	41 ‒ 47 days	Horizontal	8
Old Horizontal (OH)	6 months	Horizontal	8
Control	(See text)	No incision	9
		Total	80

Once the incision was made, only the skin was closed with continuous Mononylon 4.0 sutures, while the muscle remained open to mimic the condition of an incisional hernia. Four weeks after the procedure, the animals were euthanized and two samples of scar tissue from the muscle layer were collected from each animal. In addition, the extension of hernia regression was measured in all groups, by removing the skin of the euthanized animal and measuring the largest dimension of the scar formed at the muscle opening. During the entire time between the procedure and euthanasia, the rats were kept in controlled-environment cages, under analgesia, with water and food. The results of the measurements of groups were compared.

For histological analysis, two samples of the scar tissue were taken from each rat and kept for 24 hours in 10% formaldehyde. After fixation, the specimens were dehydrated and then embedded in paraffin; 4-μm thick sections were stained with hematoxylin-eosin and picrosirius red.

The hematoxylin-eosin staining allowed us to analyze the general morphology of the tissue. The picrosirius red staining observed under polarized light was helpful to show the structural arrangement of collagen fibers. Histologically, type I collagen present in mature fibrotic lesions is strongly birefringent, showing a yellow or red color when exposed to polarized light after staining with picrosirius red. Type III fibers present in the weaning animals’ granulation tissue, also called reticular fibers, are histologically individualized, forming thin networks with low refringence and a greenish color.

The histological characteristics analyzed in the slides stained with hematoxylin-eosin were the total number of vessels and inflammatory infiltrates. To analyze these characteristics, 5 random fields were selected per slide (under 400 × magnification). The total number of vessels was a quantitative measurement, while the analysis of inflammatory infiltrates was performed using a semi-quantitative method based on scores (0 = absent; 1 = mild; 2 = moderate; 3 = intense).

The collagen present in the analyzed fields was measured using the NIS Elements BR 3.2 software (Nikon Instruments Inc., Tokyo, Japan). A selection of red or orange birefringent shades (corresponding to type I) or greenish collagen fibers (corresponding to type III collagen fibers) was performed.^10^, ^11^, ^12^ The percentages of each type of collagen fiber present in the samples were calculated and the potential differences in these proportions between the groups were assessed.

The immunohistochemical analysis to evaluate fibroblast cell proliferation was performed using the Ki-67 antibody (rabbit anti-human ki67 monoclonal antibody - clone SP6 - SPRING Bioscience - CA). The number of immunopositive cells (stained in brown) was counted using the NIS Elements BR 3.2 software (Nikon Instruments Inc., Tokyo, Japan). Ten random fields were analyzed per slide (under 400 × magnification) and the number of immunopositive cells was quantified per field. Therefore, the final value for comparison between the groups was expressed as the number of stained cells per field.

The molecular analysis assessed the expression of the Lox gene by quantitative Real-Time PCR (qRT-PCR). This is a fluorescence technique for monitoring DNA amplification within each cycle. The number of copies of the gene was determined using Sybr Green I, a fluorescent dye that intersperses with double-stranded DNA. Fluorescence is captured by the thermocycler at each new PCR cycle reaction and allows the equipment to draw an amplification curve for each sample.

The analysis of gene amplification by qRT-PCR was performed using relative quantification, in which the gene expression of one sample is described in relation to another. In this case, samples from control animals not submitted to the procedure were used, and only muscle specimens from these animals were collected.^13^

The statistical comparison between groups was done by age (weaning, adults, and old) and by the orientation of the incision (vertical, oblique, and horizontal). In the comparison between these groups, the variables analyzed were hernia size, neovascularization, inflammatory infiltrates, percentage of immature and mature collagen, the proliferation of fibroblasts, and expression of the Lox gene.

Continuous quantitative data were analyzed using the one-way ANOVA method to compare three or more groups. Nonparametric data were analyzed using the Kruskal-Wallis test to compare three or more groups. The tests were performed using the SPSS version 18.0 for Windows (SPSS, Chicago, USA) and the equality hypothesis was rejected for *p* ≤ 0.05.

## Results

**Fig. 1:**
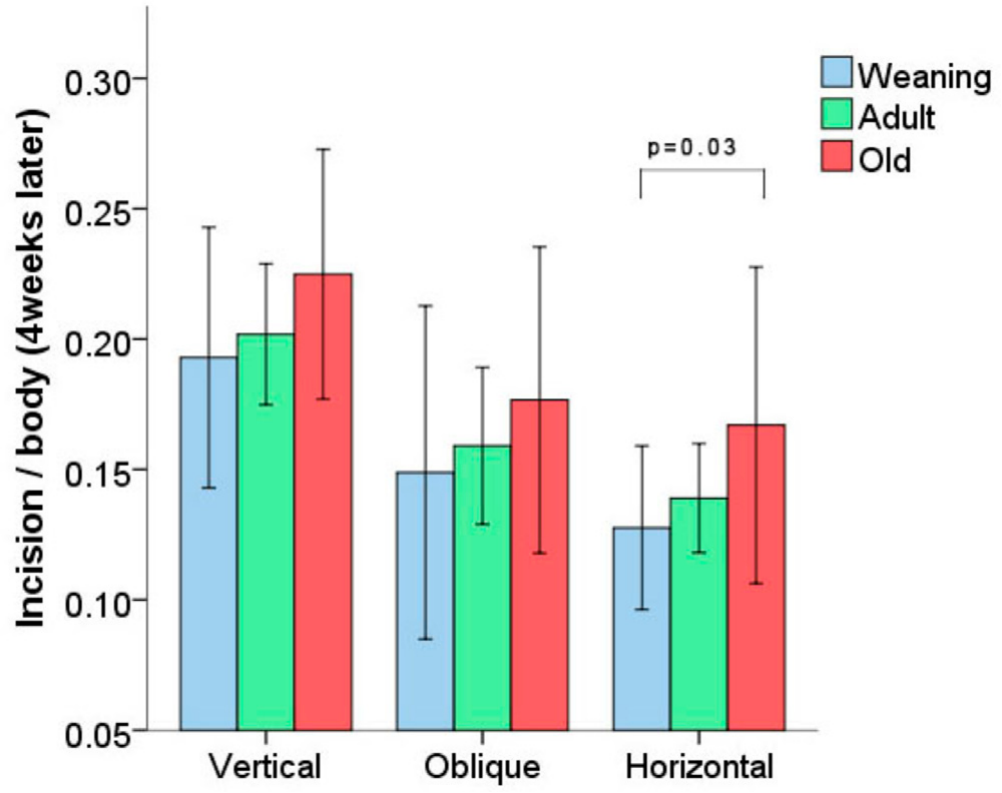
Proportion between incision size and rat size 4 weeks after surgery (mean ± SD). Vertical and oblique incisions corresponded to 25% of the animal's body length. Horizontal incisions corresponded to 20% of the animal's body length.

Hernia size analysis: in all groups of rats subjected to a specific incision (vertical, horizontal, or oblique), a reduction of the opening in the muscle created surgically was noted four weeks after the procedure. Rats of different ages subjected to the same type of incision did not present statistically significant differences regarding the reduction of the initial incision (Fig. 1). The only exception was observed in the groups subjected to horizontal incision, in which WH group animals presented a reduction of the opening greater than the OH group (*p* = 0.03), despite the fact that horizontal incisions were always proportionally smaller (20% of the size of the animal) than the vertical incisions (25% of the size).

**Fig. 2:**
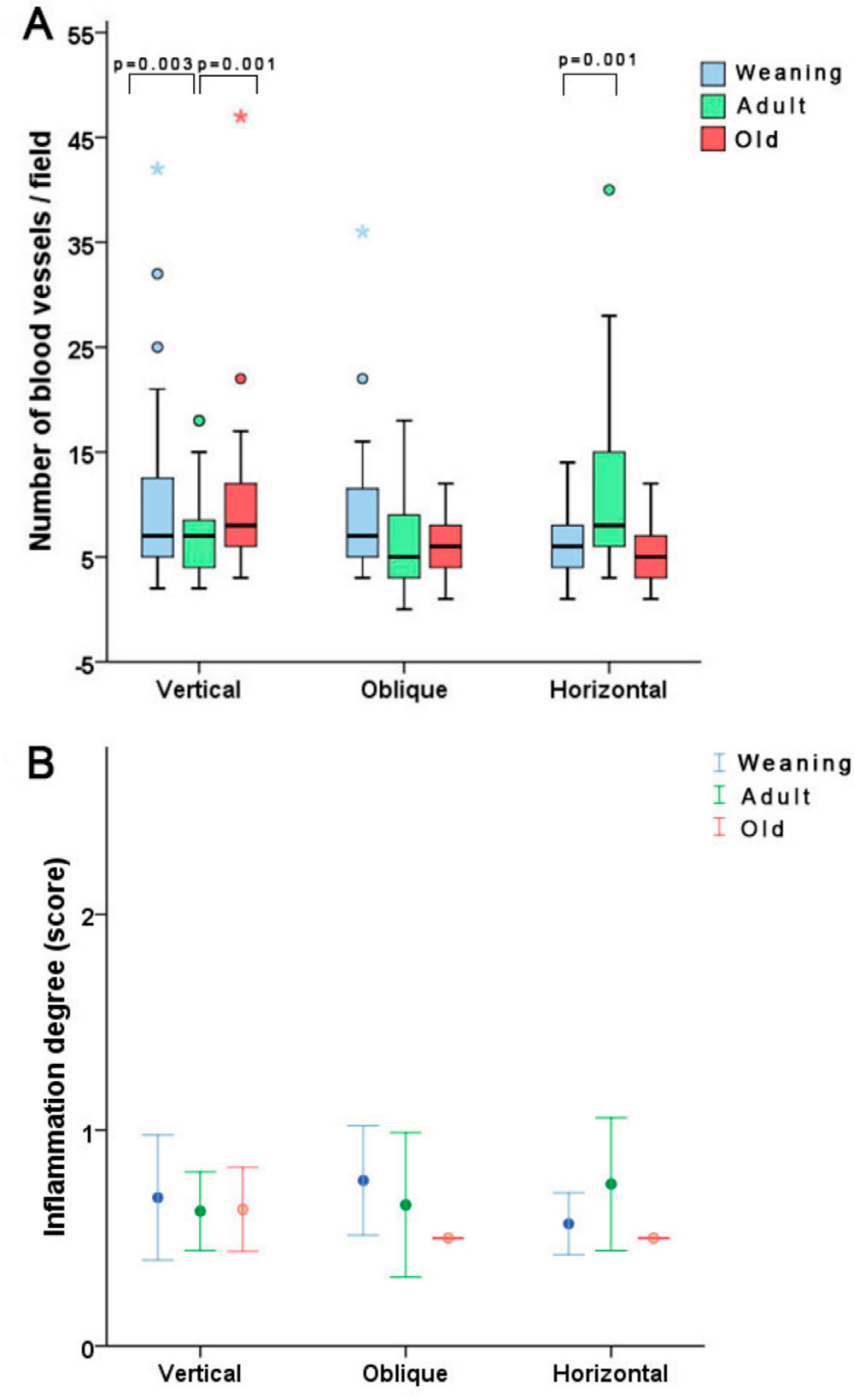
Results of histological analysis. Regarding the number of vessels per field in the groups, there were some statistical differences, although no differences were observed in the inflammation degree score. Note some outlier values.

The histological analysis revealed that concerning neovascularization, the WV group presented more blood vessels vs. the AV group (*p* = 0.003) and the AV group exhibited less neovascularization vs. the OV group (*p* = 0.001). After horizontal incision, there was a statistically significant difference between the WH and AH groups, with a higher number of new vessels found in the AH group (*p* = 0.001). Regarding the level of inflammation, there was no statistical difference between groups. (Fig. 2).

**Fig. 3:**
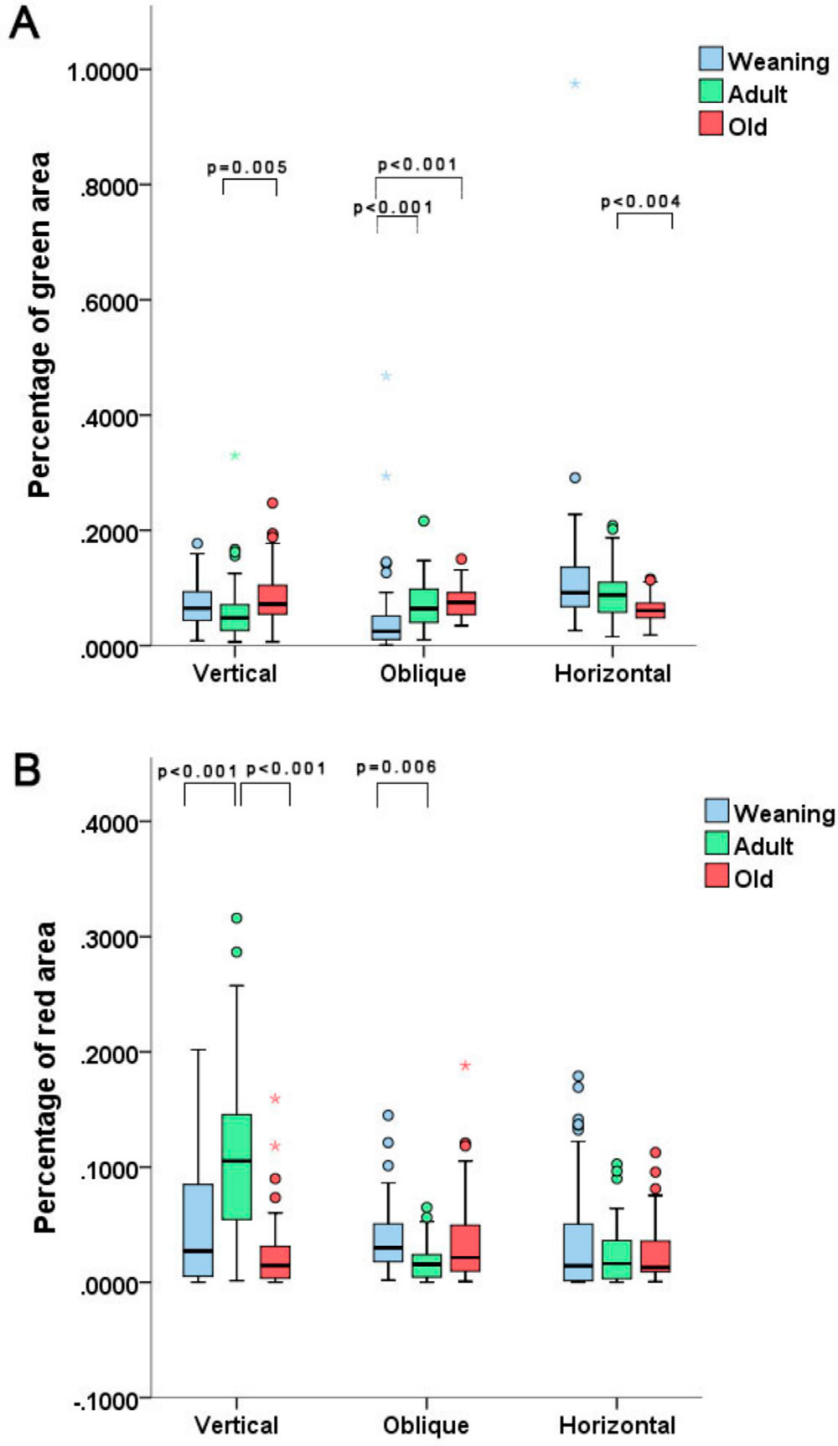
Plots showing percentage of immature collagen (A) and mature collagen (B) among the different groups. Averages tended to be similar, but there were some statistical differences. Note the outlier values.

In the collagen quantification analysis, the AV group displayed a lower percentage of immature collagen when compared to the OV group (*p* = 0.005). The WO group exhibited a lower percentage of immature collagen vs. the AO and OO groups (*p* < 0.001 in both comparisons. With horizontal incisions, the only statistically relevant difference was found between the OH and AH groups, with a higher percentage of immature collagen found in the AH group (*p* < 0.004). Concerning mature collagen, the quantification revealed a higher percentage of this protein in the AV group vs. the WV and OV groups (*p* < 0.001 in both comparisons). The WO group displayed a higher degree of mature collagen vs. the AO group (*p* = 0.006) (Fig. 3).

**Fig. 4:**
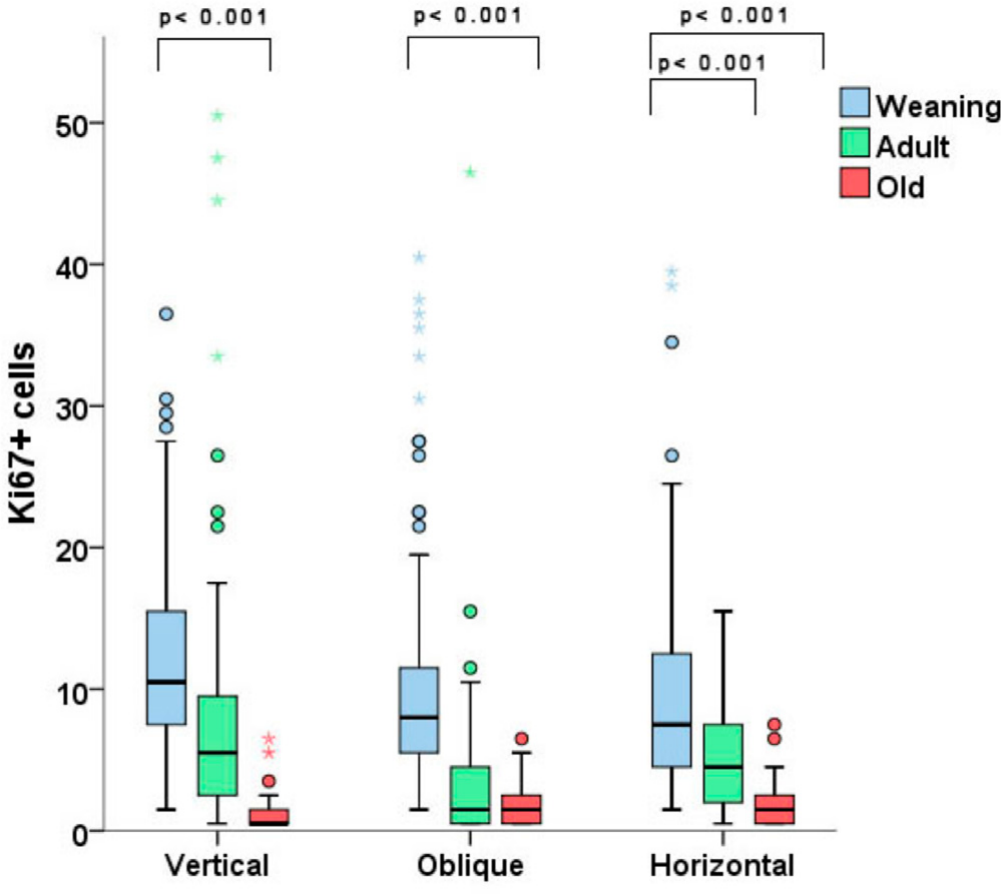
Number of ki-67-stained fibroblasts per field. There were differences in the results with each type of incision, with weaning groups showing greater proliferation of fibroblasts than older rats. Note the outlier values.

The immunohistochemical analysis showed a clear difference in fibroblast proliferation among rats of different ages, regardless of the incision orientation. With vertical incisions, the WV group exhibited a higher number of stained cells per field vs. the OV group (*p* < 0.001). With oblique incisions, the only statistically significant difference was found between the WO and the OO groups, with weaning animals presenting more ki67-stained fibroblasts. Similarly, in the horizontal incision groups, younger animals displayed more stained cells, with differences found between the WH and AH groups (more stained fibroblasts in the WH group) and between the AH and OH groups (with AH displaying more stained fibroblasts) (*p* < 0.001 for both comparisons, Fig. 4).

**Fig. 5:**
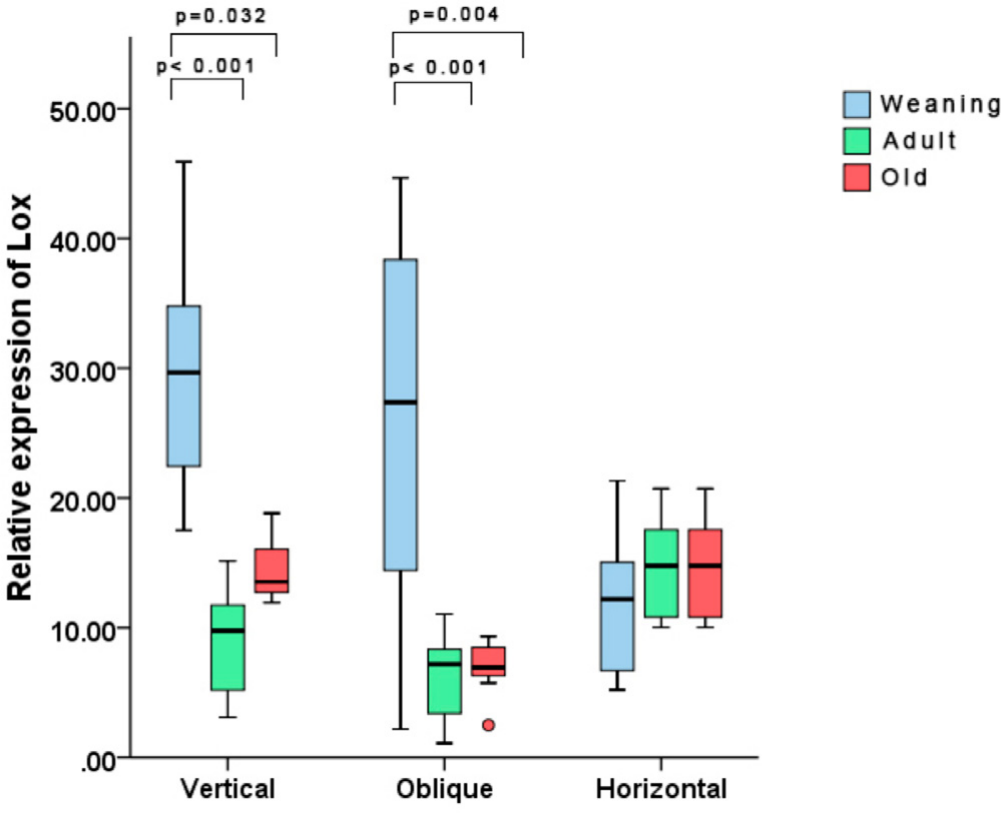
Expression of the Lox gene in different groups. The gene was more expressed in weaning rats with vertical and oblique incisions. Note one outlier value in the OO group.

Regarding the molecular analysis, a significant difference was found between the vertical and oblique incision groups, but no difference was noted among the three different age groups subjected to horizontal incisions. With vertical incisions, the WV group displayed a higher expression of the Lox gene vs the AV and OV groups (*p* < 0.001 and *p* = 0.032, respectively). With oblique incisions, a similar phenomenon occurred, with the younger WO group showing higher expression of the Lox gene vs. the AO and OO groups (*p* = 0.004 and *p* < 0.001, respectively) (Fig. 5).

## Discussion

The results of the current investigation show that certain elements present in the scar tissue may help to explain the difference in the incidence of incisional hernia observed between adult and pediatric patients. The advantage of the chosen model is the fact that the same elements are found in the scar tissue of both rats and humans, namely mature and immature collagen, fibroblasts, blood vessels, and inflammatory infiltrates. Thus, the authors can analyze these elements and infer that their behavior in rats is analogous to that of the human species. However, the differences in anatomy between rats and humans, namely the quadrupedal position, make it difficult to actually measure the hernia formed and casts doubt on whether the measurements can be extrapolated to humans.

All groups exhibited a reduction in the size of the hernia 4 weeks after the procedure, but only the horizontal incision groups presented statistically relevant differences, with shorter hernias in the WH group vs. the OH group. These results are consistent with those found in the current medical literature, i.e., a higher degree of scar tissue contraction in younger vs. older animals (rats and humans).^14^ Furthermore, other studies reveal a higher incidence of incisional hernias with vertical vs. horizontal incisions.^2^^,^^15^ The difference found between the WH and OH groups (not seen in rats subjected to the vertical procedure) may suggest that the scar tissue contraction is more noticeable in horizontal incisions than in vertical ones, explaining the lower incidence of incisional hernias with horizontal incisions.

Based on vessel quantification, younger animals subjected to vertical incisions had more intense neovascularization than adults, while among those subjected to horizontal incisions, adult animals exhibited greater neovascularization than weaning animals. This finding could be explained by the fact that the histopathological arrangement may change with age depending on the incision or by imperfections of the methods used. Some have shown increased neovascularization in the scar tissue of young rats, while other studies found a reduction in such a process.^14^ Therefore, more studies with a greater number of samples and different methodologies are needed in order to determine if neovascularization is really affected by age and by incision orientation.

The analysis of mature and immature collagen also had unexpected results. Initially, the percentage of immature collagen (green stain), was lower in the AV group vs. the OV group, yet such percentage was higher in the AH group vs. the OH group. The mature collagen analysis also displayed divergences, with a higher degree of red areas in group AV in comparison to the other groups, and a statistical difference between the WV and OV groups. However, in the oblique incision groups, the WO group had a higher degree of red areas than the AO group. Again, that could be explained by the fact that the histopathological arrangement may change with age, depending on the incision or by imperfections of the utilized methods. Nevertheless, the averages observed in the groups tended to not show statistical differences. These findings agree with the conclusions of Swift et al.^16^ since these authors stated that the amount of collagen in mature scars tends to be the same in weaning and adult rats.

The immunohistochemical analysis indicated a clear increase in fibroblast proliferation in younger animals with any type of incision. Ki-67 is a marker of cell proliferation used in this study to support the authors’ hypothesis that the staining of fibroblasts in younger animals would be more intense than in older animals.^17^ Other studies have acknowledged the importance of fibroblast proliferation in the formation of incisional hernias, such as the one from Franz et al.^18^ that quantified the number of Ki-67-stained fibroblasts in cicatricial tissue from hernia sacs of rats in 3 different postoperative stages; a limitation of this study was the absence of comparison of fibroblast proliferation between rats of different ages. In an interesting study by Ryzhak et al., specimens from younger animals displayed a greater degree of Ki-67 staining vs. old rats in cells from the retina, testicle and kidney subjected to in vitro proliferation.^19^ These findings are consistent with those observed in the present study, which shows a higher proliferative capacity in weaning rats when compared to adult ones and in adult ones when compared to older animals. Therefore, the intense proliferative capacity of different cell types, especially fibroblasts in cicatricial tissue demonstrated in younger rats, could help to explain why incisional hernias are less prevalent in the pediatric population when compared to adults. Finally, it is important to stress that healing wounds also respond to the functional demands of the mechanical environment and physical forces, although an understanding of the fundamental mechanism by which mechanical stress affects tissues and wounds remains elusive.^20^

In the current investigation, the authors studied a gene that encodes a member of the lysyl oxidase family of proteins (Lox gene). A higher expression of this gene was observed in younger rats subjected to vertical and oblique incisions. Until now, no association was found between this gene and the cicatricial tissue of incisional hernias, even though in some studies an intense expression of Lox was observed in the extracellular matrix remodeling after injury to skin tissues.^8^ The lysyl oxidase protein is known to help stabilize the matrix during its formation, development, maturation, and remodeling.^9^ Considering its direct action on the collagen and extracellular matrix metabolism after tissue injury, the authors aimed to evaluate the expression of the Lox gene in the cicatricial tissue of rats at different ages, with a higher expression being found in younger groups. This phenomenon supports the hypothesis that this gene could be associated with more efficient healing in the pediatric population, which may explain the lower incidence of incisional hernias among children.

In conclusion, the current study found that smaller hernias after horizontal incisions, higher fibroblast proliferation, and higher Lox gene expression in weaning rats were important differences between the younger and older animals. Such findings could be associated with a lower incidence of incisional hernias in children when compared to older individuals. The literature still lacks comparative studies of scar tissue in different age groups which might corroborate the conclusions of this study.

## Abbreviations

Adult Horizontal (AH); Adult Oblique (AO); Adult Vertical (AV); Old Horizontal (OH); Old Oblique (OO); Old Vertical (OV); Quantitative Real-Time PCR (qRT-PCR); Weaning Horizontal (WH); Weaning Oblique (WO); Weaning Vertical (WV)

## Authors' contributions

Rafael Nogueira do Amaral, Ana Cristina Aoun Tannuri, Junia Marielle Teixeira Rodrigues Neri, and Hugo de Souza Reis performed the animal experiments; Josiane de Oliveira Gonçalves, Suellen Serafini performed all laboratory studies and the statistical analyses; Uenis Tannuri was responsible for the final approval of the investigation and for the manuscript revision. All authors have read and approved the manuscript.

## Funding

The project had financial support from 10.13039/501100001807Fundação de Amparo à Pesquisa do Estado de São Paulo, process number 2019/10,347–4.

## Conflicts of interest

The authors declare no conflicts of interest.
